# UHF Partial Discharge Location in Power Transformers via Solution of the Maxwell Equations in a Computational Environment

**DOI:** 10.3390/s19153435

**Published:** 2019-08-05

**Authors:** Luiz A. M. M. Nobrega, Edson G. Costa, Alexandre J. R. Serres, George V. R. Xavier, Marcus V. D. Aquino

**Affiliations:** Department of Electrical Engineering, Universidade Federal de Campina Grande, Aprigio Veloso 882, Universitário, Campina Grande 58429-900, Brazil

**Keywords:** partial discharge, power transformer, UHF, sensor, monitoring, localisation, binary particle swarm optimisation

## Abstract

This paper presents an algorithm for the localisation of partial discharge (PD) sources in power transformers based on the electromagnetic waves radiated by a PD pulse. The proposed algorithm is more accurate than existing methods, since it considers the effects of the reflection, refractions and diffractions undergone by the ultra-high frequency (UHF) signal within the equipment tank. The proposed method uses computational simulations of the electromagnetic waves generated by PD, and obtains the time delay of the signal between each point in the 3D space and the UHF sensors. The calculated signals can be compared with the signals measured in the field, so that the position of the PD source can be located based on the best correlation between the simulated propagation delay and the measured data. The equations used in the proposed method are defined as a 3D optimisation problem, so that the binary particle swarm optimisation algorithm can be used. To test and demonstrate the proposed algorithm, computational simulations were performed. The solutions were sufficient to identify not only the occurrence of defects, but also the winding and the region (top, centre or base) in which the defect occurred. In all cases, an accuracy of greater than 15 cm was obtained for the location, in a 180 MVA three-phase transformer.

## 1. Introduction

Power transformers are among the most important components of an electrical power system, due to their high cost and functionality within the system. Monitoring of the levels of partial discharge (PD) activity inside transformers is an important step in the predictive maintenance process, as it can indicate internal faults that must be corrected before the system is compromised.

Several techniques have been used to detect PD within a transformer, such as the standard method defined by IEC 60270 [1], dissolved gas analysis (DGA) [2,3,4], the acoustic method [5,6,7] and the ultra-high frequency (UHF) method [8,9,10,11,12]. In addition to PD detection, studies have been carried out to determine the location of the PD based on the acoustic wave generated by the PD or the electromagnetic radiation in the UHF band. However, the acoustic method has less sensitivity to low-intensity PD and to those occurring within the winding, so the UHF method is therefore preferable [5,13,14,15].

UHF location of PD in power transformers has been traditionally performed using the time difference of arrival (TDOA) between signals that are captured from a set of UHF sensors. From these, the PD location can be found by geometric triangulation, which involves solving a set of nonlinear equations. Two approaches have been used. The first is the traditional method, which presupposes a line-of-sight propagation from the source to the sensors, so that the location of the PD source can be performed based on the solution of simple distance equations [16,17,18]. However, this assumption induces errors in the PD location by neglecting the obstacles in the propagation path. The second approach involves the calculation of the signal propagation time based on geometric modelling of the power transformer, followed by an algorithm that correlates the calculated values with the measured data [19,20]. Although the use of geometric modelling results in a better PD location estimation than the straight line-of-sight method, the geometric method gives a very simplified model of the transformer and is not able to represent all of the effects along the propagation path, such as reflections, refractions and diffractions in different materials, and this causes errors in the TDOA estimation.

In order to optimise the PD location, a third approach can be used that corresponds to estimation of the TDOA from the solution of the Maxwell equations in a computational environment. As shown in [21], the estimated propagation times for the signals based on computational simulations are closer to the experimental results than when using simple distance equations or geometric modelling. In addition, computational simulation of the UHF PD propagation using the Maxwell equations shows reasonable agreement with PD phenomena measured in the laboratory [21].

The objective of this paper is to propose a PD localisation algorithm for power transformers that uses the solution of Maxwell equations in a computational environment to estimate the TDOA between signals captured from a set of UHF sensors. Using the proposed algorithm, it is possible to include the reflection, refraction and diffraction effects that occur during the propagation path in the localisation method.

The rest of this paper is organised as follows: Section 2 explains the localisation algorithm. Detailed simulations and the 3D modelling technique for testing and demonstrating the localisation algorithm are described in Section 3. In Section 4, the results of the computational procedures are presented and discussed. Section 5 presents the conclusions.

## 2. PD Localisation Method

This work proposes a method for the location of PD in power transformers, based on the solution of the Maxwell equations in a computational environment. Four procedures are used, as described below.

### 2.1. Sensor Positioning

**Figure 1:**
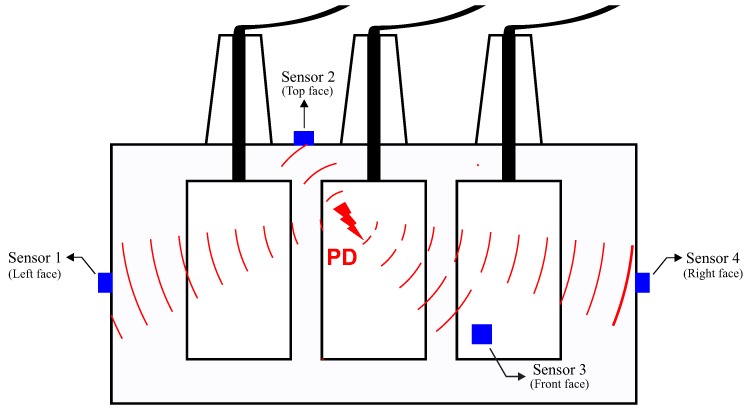
Positions of the ultra-high frequency (UHF) sensors.

Initially, at least four UHF sensors must be installed in the equipment tank through dielectric windows, as previously described in [8]. The sensors must be installed with the maximum spatial distance between them on the surface of the transformer tank and not geometrically placed on the same plane [10], as illustrated in Figure 1.

### 2.2. Transformer Modelling

**Figure 2:**
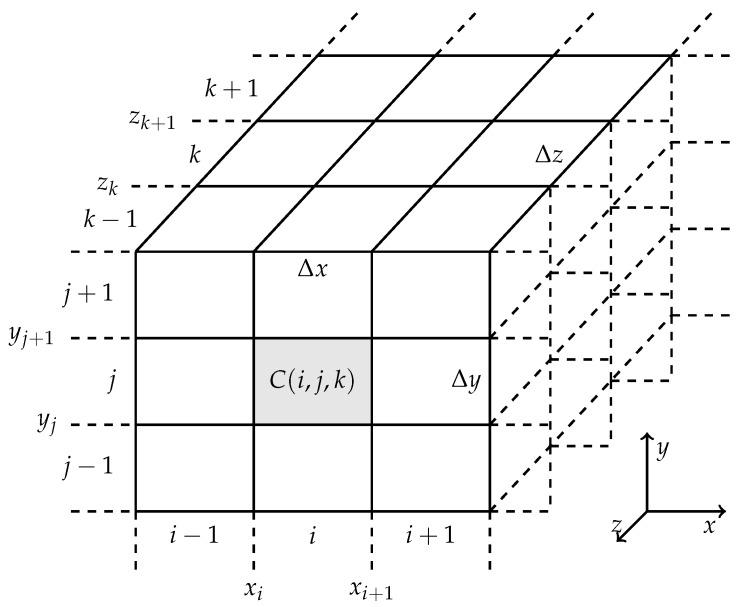
Representation of the 3D matrix of coordinates.

The second procedure corresponds to the modelling of the power transformer. For this, the power transformer must first be modelled in software that allows 3D electromagnetic simulation. A 3D matrix composed of cells of size Δx×Δy×Δz is then created in order to represent the various positions in the transformer model, where each point in the 3D space is represented by the x=iΔx, y=jΔy and z=kΔz coordinates [19], as illustrated in Figure 2.

### 2.3. Calculation of the Propagation Matrix

**Figure 3:**
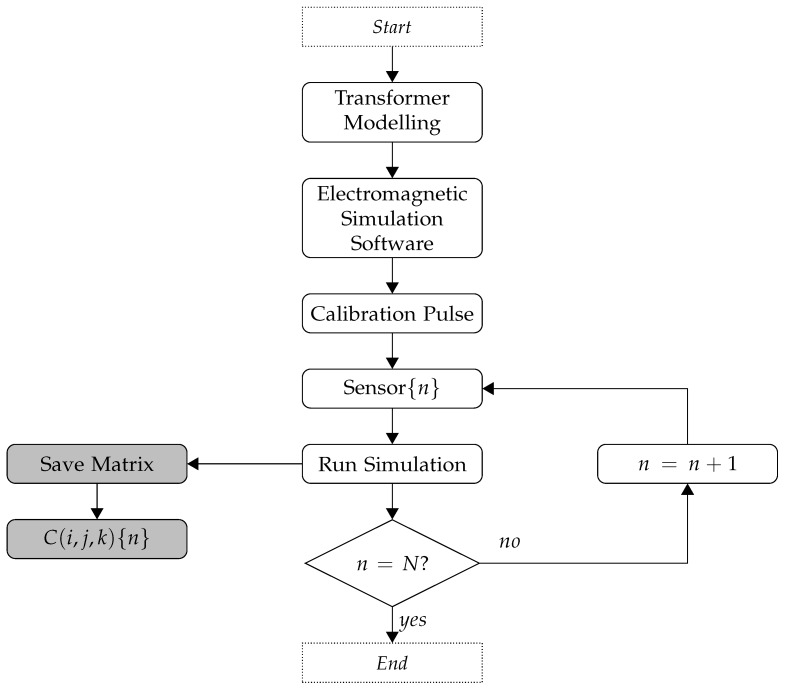
Flowchart of the proposed methodology to obtain the transformer propagation times.

Once the discrete transformer model has been created, as represented by the matrix C(i,j,k), the third step is the calculation of the propagation time for a UHF signal from each cell in the 3D space to the UHF sensors on the equipment tank. Figure 3 shows a flowchart of the proposed methodology for obtaining the propagation times, generalized to *N* sensors. Initially, a calibration pulse is injected from one of the sensors n={1,2,…,N} into the simulator, and the signal propagation time $\Delta t_{n}^{C}$ is recorded for each model cell C(i,j,k). The procedure is then repeated by changing the origin of the calibration pulse to each of the other sensors. The equivalent of *N* computational simulations are performed and *N* matrices C(i,j,k){n} are obtained with the propagation times.

The calculation of the propagation time is based on the Fermat principle. According to this principle, in an isotropic environment, the signal time for propagation from point A to point B is the same as for propagation from point B to point A. Thus, instead of simulating the signal propagation from all points of the mesh C(i,j,k) to the *N* sensors, which would demand a large computational effort, the signal propagation is simulated from the sensors to all points of the mesh. The procedure is performed only once for each transformer geometry, and this is an advantage of the proposed method. The model can then be used for continuous, online and permanent monitoring of the transformer by means of the fourth procedure described below.

### 2.4. Binary Particle Swarm Optimisation (BPSO) Algorithm for 3D Optimisation

Once the propagation matrix C(i,j,k){n} has been obtained for each sensor *n*, the location of PD can be carried out for the modelled transformer. The TDOA of the signals to sensors 1 to *N* are measured and the PD location is then performed by determining the cell that minimises the following objective function, as proposed in [20]:

(1) $$cost\left(C_{i,j,k}\right)=\sqrt{{\left(\Delta t_{12}^{m}-\Delta t_{12}^{C}\right)}^{2}+{\left(\Delta t_{13}^{m}-\Delta t_{13}^{C}\right)}^{2}+\ldots +{\left(\Delta t_{1N}^{m}-\Delta t_{1N}^{C}\right)}^{2}},$$

where the superscripts *m* and *C* refer to the measured and calculated times for cell $C_{i,j,k}$, respectively. In other words, the location method consists of a search by the cell in space (or set of cells) where the time delays correspond to the measured data within a specified tolerance. In order to obtain the minimum value of Equation (1), it is proposed to use the binary particle swarm optimisation (BPSO) algorithm, instead of the conventional particle swarm pptimisation (PSO) proposed in [20], since the binary version of the method is more appropriate for discrete optimisation.

## 3. Evaluation of the Localisation Algorithm

In order to test the proposed algorithm, computational simulations were used to represent the PD phenomenon in power transformers. The following procedures were adopted: (*i*) A real power transformer was modelled using electromagnetic simulation software, (*ii*) PD were simulated in the transformer windings and the signals were received from four simulated UHF sensors located on the equipment tank, and (*iii*) a demonstration test of the proposed localisation algorithm was performed.

### 3.1. Construction Characteristics of the Modelled Transformer

To test the algorithm, 3D modelling of a 230/69 kV, 180 MVA three-phase transformer was performed. Table 1 shows the characteristics of each element of the modelled transformer. As can be seen, the number of turns and the gaps between the winding discs for radial oil ventilating were considered.

### 3.2. Modelling in CST Microwave

The propagation of PD-related EM waves was simulated using the finite integration technique (FIT) through the application of CST Microwave Studio software. The FIT is a discrete method that solves Maxwell’s equations in an integral form rather than the differential form used in the finite-difference time-domain (FDTD) method [22]. However, other software that can give a solution to the Maxwell equations could also have been used.

**Figure 4:**
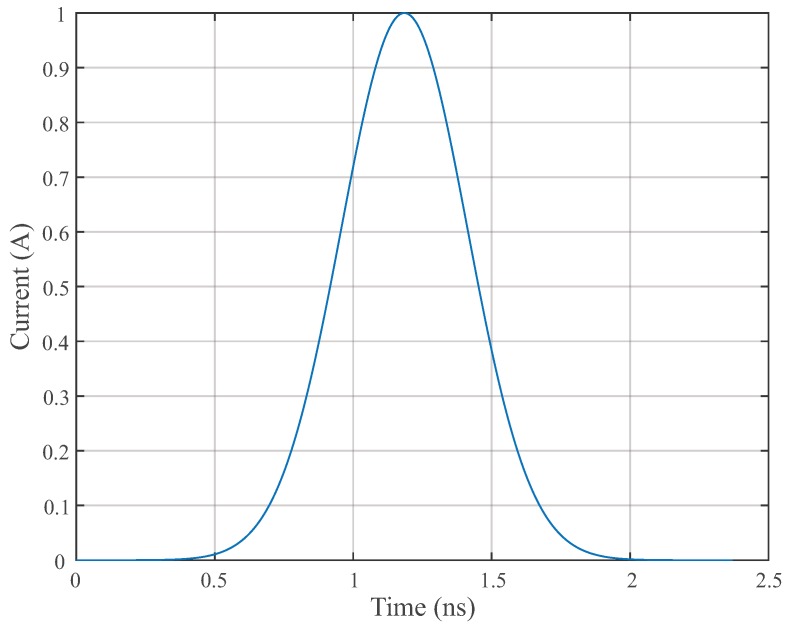
The Gaussian PD current wave.

A Gaussian pulse of width of 1.5 ns, current 1 A, and radiated UHF frequency in the range 0 to 1.5 GHz was used to represent the PD phenomenon [20,23,24,25]. Figure 4 shows the Gaussian pulse used to represent the PD pulse.

In order to reduce the computational effort, certain simplifications were adopted in the power transformer model based on recommendations found in the literature. All of the metallic structures were modelled as perfect electric conductors (PEC) [23], and the insulating paper present in the conductors and windings was neglected [20,26]. With these simplifications, the computational effort was markedly reduced, with negligible effect on the PD propagation. The propagation was simulated in transformer oil with a relative permittivity of 2.33.

### 3.3. Simulation of Defects

**Figure 5:**
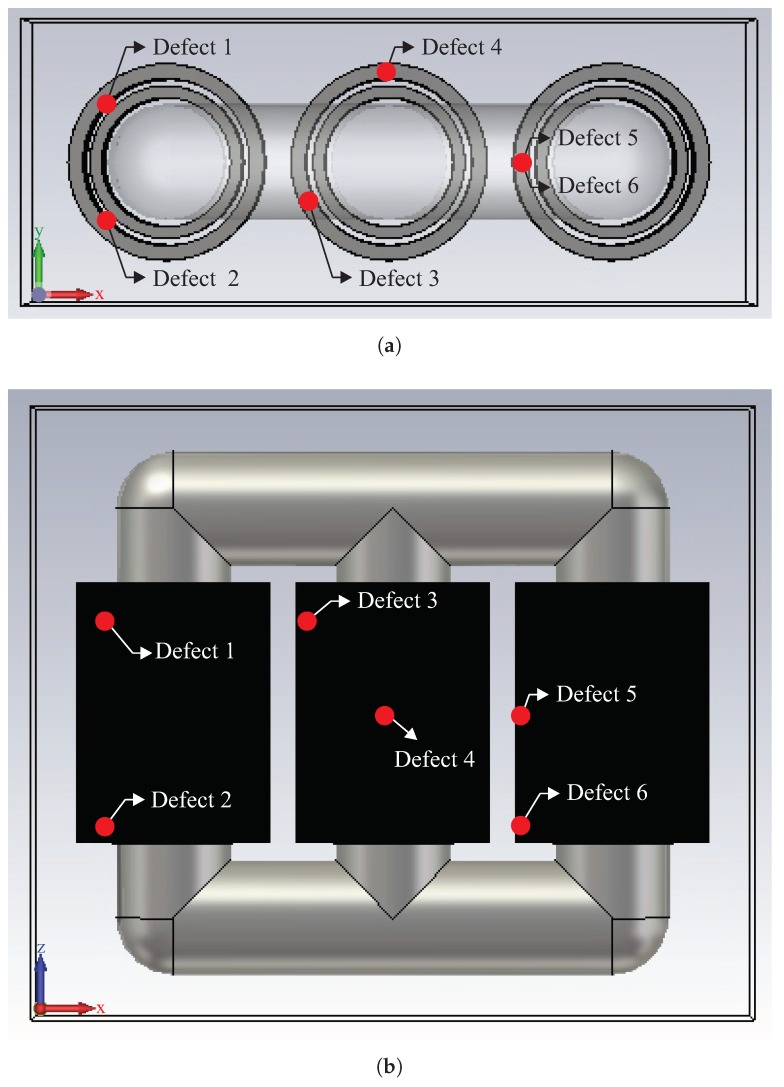
Position of simulated defects: (**a**) Top view of the simulated transformer, and (**b**) front view of the simulated transformer.

Six 3D simulations were created to evaluate the proposed localisation algorithm, and in each case, the defect was simulated in a different position inside the equipment. Priority was given to locating defects in various positions, including the three phases and positions at the top, centre and base of the windings. Figure 5 shows a graphical representation of the transform with the positions of the simulated defects.

**Figure 6:**
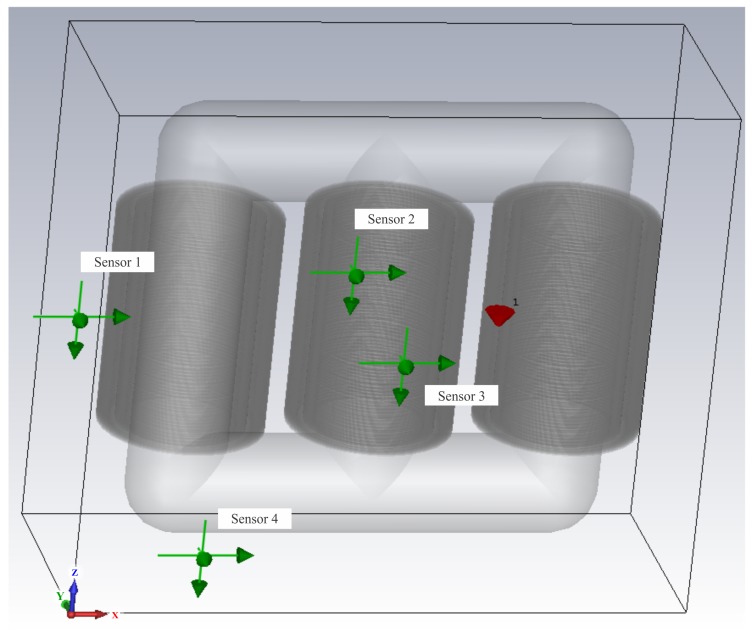
Model used to test the localisation algorithm.

Once the positions of the defects were chosen, the sensors were located in the simulation. The sensors were modelled as an electric field probe, and were arranged in four distinct positions as shown in Figure 6. The coordinates of both the defects and the sensors for each case simulated here are given in Table 2, for a coordinate system centred on the lower left corner of the transformer.

To apply the localisation algorithm, we first obtained the propagation matrix C(i,j,k) for the transformer under analysis, according to the procedure set out in Section 2. The localisation algorithm was then applied in the six situations described above, using the BPSO algorithm to minimize Equation (1). Since the BPSO algorithm is a probabilistic method, it was applied twenty times, and the centroid of the solutions obtained at each iteration was taken as the global solution of the proposed method.

The simulations using the CST Microwave were performed in approximately 12 h each, using a 64,473,750 meshcell on an Intel Xeon E5-2620 computer with 12 processing cores of 2 GHz and 128 GB of DDR3 memory. The BPSO algorithm parameters were an inertia weight of 1.1 and learning coefficients of 1.49. One hundred particles were used, and were distributed randomly within the equipment. A common personal computer was used for the BPSO application.

## 4. Results

The results obtained in this work are presented below.

### 4.1. Transformer Propagation Model

**Figure 7:**
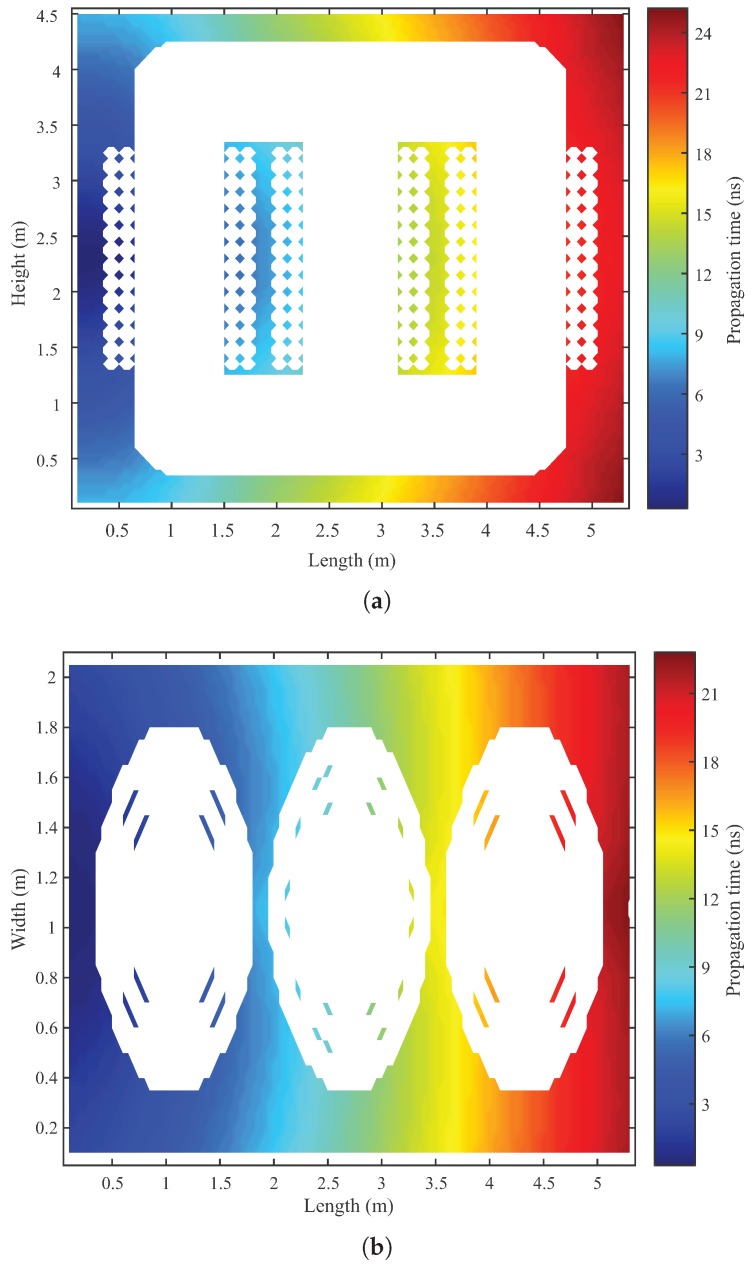
Signal propagation time to the sensor located on the left side of the equipment tank (sensor 1): (**a**) Longitudinal section along the central axis of the transformer, and (**b**) horizontal section along the central axis of the transformer.

The construction of the transformer propagation model is the first step of the localisation algorithm. As explained in Section 2, this model needs to be obtained only once, and can be used as many times as necessary in the localization algorithm. In order to graphically represent the obtained matrix C(i,j,k) from the transformer under analysis, a longitudinal and horizontal cross-section along the central axis of the transformer is used, as shown in Figure 7. The colours represent the propagation time of the signal from the various points in matrix C(i,j,k) to sensor 1, located on the left side of the equipment tank. The blank silhouette represents the metal structure of the transformer, where there is no signal propagation.

Figure 7 shows the possibility of obtaining the propagation times from all coordinates of the transformer to the UHF sensor. Thus, it is demonstrated that the transformer propagation model was successfully acquired by the presented methodology. It can be observed from the Figure 7 that there are regions closer to the UHF sensor that have a longer propagation time than more distant regions. This difference is explained by the fact that in some propagation paths, the signal encounters obstacles that delay it. Thus, the signal propagation time is defined not by the spatial distance between two points, but by the real propagation path that the signal takes as it circumvents the obstacles.

### 4.2. Signals Obtained from the UHF Sensors

**Figure 8:**
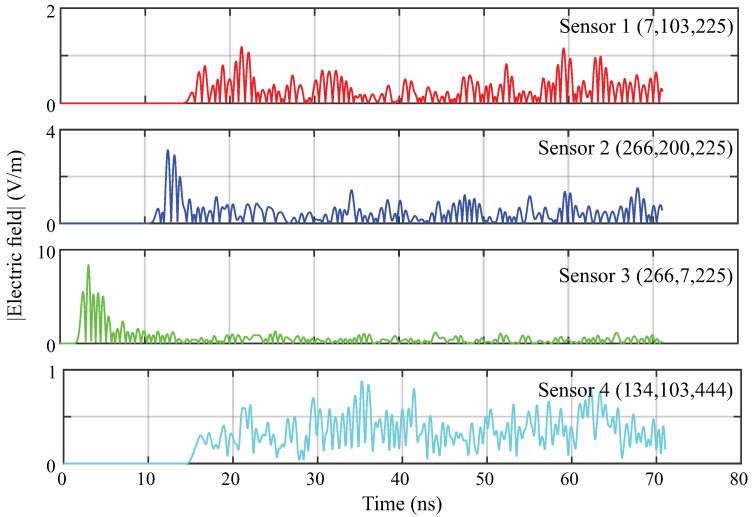
Magnitude of the signals obtained from the UHF sensors for the defect 6.

To exemplify the signals obtained from the simulations, Figure 8 shows the magnitude of the signals obtained from the sensors for the defect 6. The obtained result is similar to the acquisition of signals in a field test through the use of an oscilloscope, and can therefore be used for testing of the localisation algorithm. As observed, the signals show different intensities and propagation times for each sensor; this is as expected, since the signals travel along different paths to the installed UHF sensors. Calculating the propagation time for the case presented below gives times of 14.25 ns, 10.21 ns, 1.4 ns and 14.37 ns for sensors 1 to 4, respectively.

### 4.3. Localisation of PD Source

**Figure 9:**
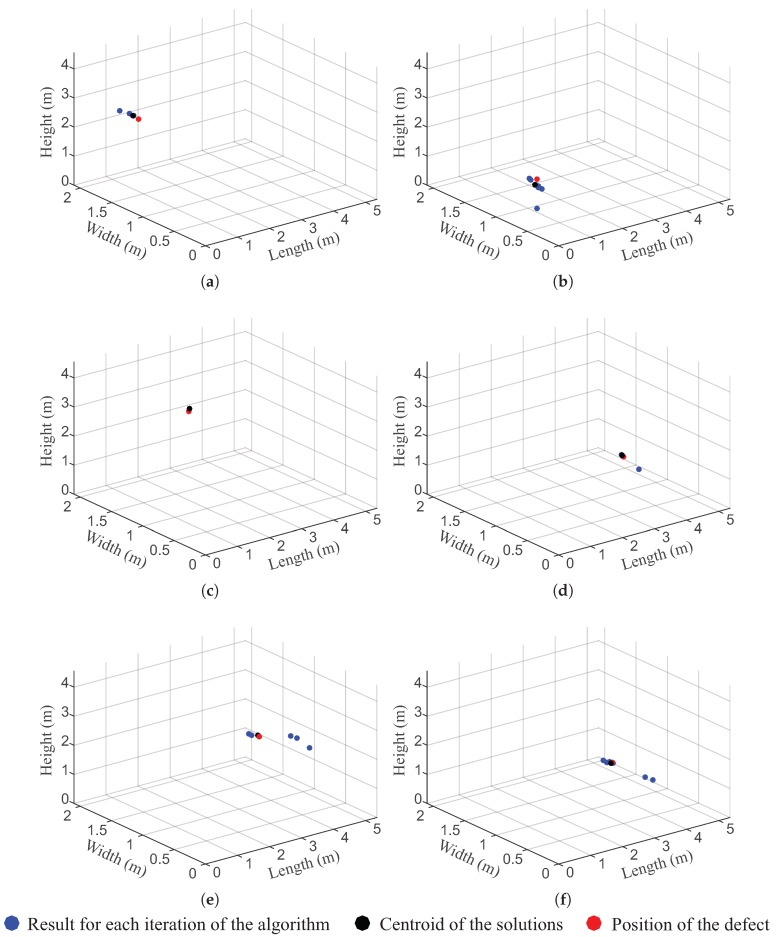
Results of the localisation algorithm: (**a**) Defect in position 1, (**b**) defect in position 2, (**c**) defect in position 3, (**d**) defect in position 4, (**e**) defect in position 5, and (**f**) defect in position 6.

From the signals obtained by the UHF sensors and the propagation matrix C(i,j,k) obtained for the transformer under analysis, the PD source was located for the six simulated cases. The solutions from the localisation method and the errors obtained are presented in Table 3 for each case, and Figure 9 illustrates these results. The blue circles indicate the 3D location obtained for each iteration of the location algorithm, the black circle indicates the geometric centre of the solutions, and the red circle indicates the position of the defect.

For the six simulated cases, we verified that the method achieved a high level of accuracy, thus meeting the demands of practical engineering scenarios. In the best case, the method had an accuracy of 4 cm in the location (defect 2), while in the worst case, the accuracy obtained was 15 cm, which is a good result considering the size of the simulated transformer. This precision was sufficient to identify not only the occurrence of defects, but also the winding in which they occurred and the region (top, centre or base) in which they were located. This information is useful for diagnosis of the equipment, since it allows us to evaluate the location and severity of the problem and facilitates a more detailed investigation of the equipment. Moreover, the convergence time of the localisation algorithm was approximately 50 s (i.e., when the stop criteria were met). Thus, the computational speed of the method is sufficiently high for all practical purposes, including online monitoring.

## 5. Conclusions

This paper has presented a new UHF PD localisation algorithm for power transformers by solving the Maxwell equations in a computational environment. The propagation delay of the signal from each point in the 3D space to the UHF sensors was first obtained, and the simulated propagation delays were then compared with the measured signals, meaning that the PD location corresponds to the position where the simulated propagation time best approaches the measured data. The equations used in the method were defined as a three-dimensional optimisation problem, so that the BPSO algorithm could be used to minimise the objective function of the algorithm.

The effectiveness of the localisation algorithm was demonstrated using six 3D simulations (with four other simulations that obtained the propagation times used in the method). The proposed algorithm was able to include the effects of the reflections, refractions and diffractions undergone by the UHF signal in the equipment tank. Thus, a methodological progress was obtained in PD location methods. In the simulated cases used for testing the algorithm, the solution was able to identify not only the occurrence of defects, but also the winding and the region (top, centre or base) in which the defect occurred. In most cases, an accuracy of greater than 15 cm was obtained for the location of the simulated defect.

Our localisation method therefore proved to be useful for diagnosis of the equipment, since it allows us to evaluate not only the severity of the problem, but also the location, and facilitates a more detailed investigation of the equipment.

## Figures and Tables

**Table 1:** Characteristics of the simulated transformer.

Parameter	Value	Parameter	Value
Rated power (MVA)	180	Gap between winding discs (mm)	5
Number of phases	3	Length of core (mm)	4095
Number of turns	127	Height of core (mm)	3870
Voltage (kV/kV)	230/69	Width of core window (mm)	780
Height of windings (mm)	1915	Height of core window (mm)	2230
Inner radius of windings (mm)	1219	Length of tank (mm)	5321
Outer radius of windings (mm)	1431	Height of tank (mm)	4500
Height of winding discs (mm)	10	Width of tank (mm)	2071

**Table 2:** Positions of sensors and partial discharge (PD) sources.

Sensor	Position (*x*, *y*, *z*) (cm)	Simulated Case	Position (*x*, *y*, *z*) (cm)
Sensor 1	(7, 103, 225)	Defect 1	(55, 135, 285)
Sensor 2	(266, 200, 225)	Defect 2	(60, 65, 145)
Sensor 3	(266, 7, 225)	Defect 3	(210, 133, 299)
Sensor 4	(134, 103, 444)	Defect 4	(265, 32, 225)
		Defect 5	(370, 103, 225)
		Defect 6	(370, 103, 135)

**Table 3:** Results obtained from the localisation algorithm.

Simulated Case	Algorithm (*x*, *y*, *z*) (cm)	Source (*x*, *y*, *z*) (cm)	Error (*x*, *y*, *z*) (cm)	Absolute Error (cm)
Defect 1	(58, 147, 286)	(55, 135, 285)	(3, 12, 1)	12
Defect 2	(55, 64, 131)	(60, 65, 145)	(−5, −1, −14)	15
Defect 3	(215, 135, 305)	(210, 133, 299)	(5, 2, 6)	8
Defect 4	(265, 35, 230)	(265, 32, 225)	(0, 3, 5)	6
Defect 5	(374, 104, 223)	(370, 103, 225)	(4, 1, −2)	4
Defect 6	(365, 107, 133)	(370, 103, 135)	(−5, 4, −2)	6
